# Giant Cell Tumor of the Distal Ulna in an Elderly Patient on Hemodialysis: Diagnostic Challenge and Surgical Management

**DOI:** 10.7759/cureus.115482

**Published:** 2026-08-31

**Authors:** Abdessabour Sebbar, Noaim Boulaich, Achraf Tebbaa El Hassali, Hicham Yacoubi, Najib Abdeljaouad

**Affiliations:** 1 Orthopedics and Traumatology, Mohammed VI University Hospital, Oujda, MAR; 2 Faculty of Medicine and Pharmacy, Mohammed First University, Oujda, MAR

**Keywords:** brown tumor, darrach procedure, distal ulna, flexor carpi ulnaris tenodesis, giant cell tumor, hemodialysis, secondary hyperparathyroidism, wrist tumor

## Abstract

Giant cell tumor of bone is a locally aggressive tumor that usually affects the epiphyseal region of long bones in young adults. Involvement of the distal ulna is rare, and the diagnosis in elderly patients with end-stage chronic kidney disease may be difficult because brown tumors related to secondary hyperparathyroidism can mimic giant cell tumor radiologically and histologically. We report the case of a 69-year-old woman with end-stage chronic kidney disease on hemodialysis who was admitted for a painful and progressively enlarging mass on the ulnar border of the left wrist that had been present for six months. Standard radiographs showed a well-defined, expansile osteolytic lesion of the distal ulna involving the epiphysis and metaphysis, with cortical thinning and no obvious periosteal reaction. Surgical biopsy demonstrated a proliferation of mononuclear stromal cells associated with numerous osteoclast-like multinucleated giant cells, compatible with a giant cell tumor. Given the hemodialysis background, a brown tumor was discussed; however, serum calcium, phosphate, and parathyroid hormone levels were within normal limits, and histology favored a giant cell tumor. The extension workup showed no secondary localization. The patient underwent en bloc resection of the distal ulna according to a Darrach-type procedure, associated with flexor carpi ulnaris tenodesis to improve the stability of the ulnar stump. Histopathological examination of the surgical specimen confirmed the diagnosis. At eight months of follow-up, the outcome was favorable, with satisfactory wrist function, a stable radiographic appearance, and no clinical or radiological recurrence. This case highlights the importance of clinicobiological and histopathological correlation when evaluating a giant-cell-rich bone lesion in a dialysis patient. It also supports the value of distal ulna resection associated with soft-tissue stabilization as a pragmatic option in selected elderly patients.

## Introduction

Giant cell tumor of bone is an intermediate, locally aggressive primary bone tumor characterized by neoplastic mononuclear stromal cells associated with osteoclast-like multinucleated giant cells. It typically occurs after skeletal maturity and favors the epiphyseal or meta-epiphyseal regions of long bones, particularly around the knee and distal radius [1-4]. Although histologically benign in most cases, it may cause cortical expansion, local recurrence, and, rarely, pulmonary metastases [3,4].

Distal-ulna localization is uncommon, and available evidence consists mainly of case reports and small retrospective series [5,6]. This location creates a specific therapeutic problem because adequate local control must be balanced against distal radioulnar stability, grip strength, pronation-supination, and avoidance of painful instability or impingement of the residual ulnar stump [6-11].

The diagnostic problem is amplified in chronic kidney disease. Brown tumors secondary to hyperparathyroidism may present as expansile osteolytic, giant-cell-rich lesions and mimic giant cell tumor on imaging and histology [12,13]. Distinguishing these entities is clinically important because their mechanisms and management differ. The combination of an uncommon distal-ulnar location, advanced age, and chronic hemodialysis therefore represents an unusual diagnostic setting.

The need for this report arises from the limited literature describing distal-ulna giant cell tumor in elderly dialysis patients and the challenge of selecting treatment that provides local control while preserving useful wrist function. We present a single-patient clinical case report describing the diagnostic workup, surgical management, and eight-month clinical and radiographic outcome after en bloc distal-ulna resection with flexor carpi ulnaris tenodesis.

## Case presentation

This is a single-patient clinical case report. A 69-year-old woman with end-stage chronic kidney disease on maintenance hemodialysis was referred for a progressively enlarging mass on the ulnar aspect of the left wrist. Symptoms had evolved for approximately six months, with progressive enlargement, persistent pain, and increasing limitation of wrist motion.

On examination, a firm, fixed mass measuring approximately 4 cm was palpable over the distal ulna. The skin was healthy, without inflammatory signs. Flexion and extension were clinically reduced because of pain and the mechanical effect of the mass. Distal neurovascular examination was normal. The available clinical record did not contain prospectively collected quantitative goniometric measurements or a standardized wrist outcome score. Figure 1 shows the preoperative appearance.

**Figure 1:**
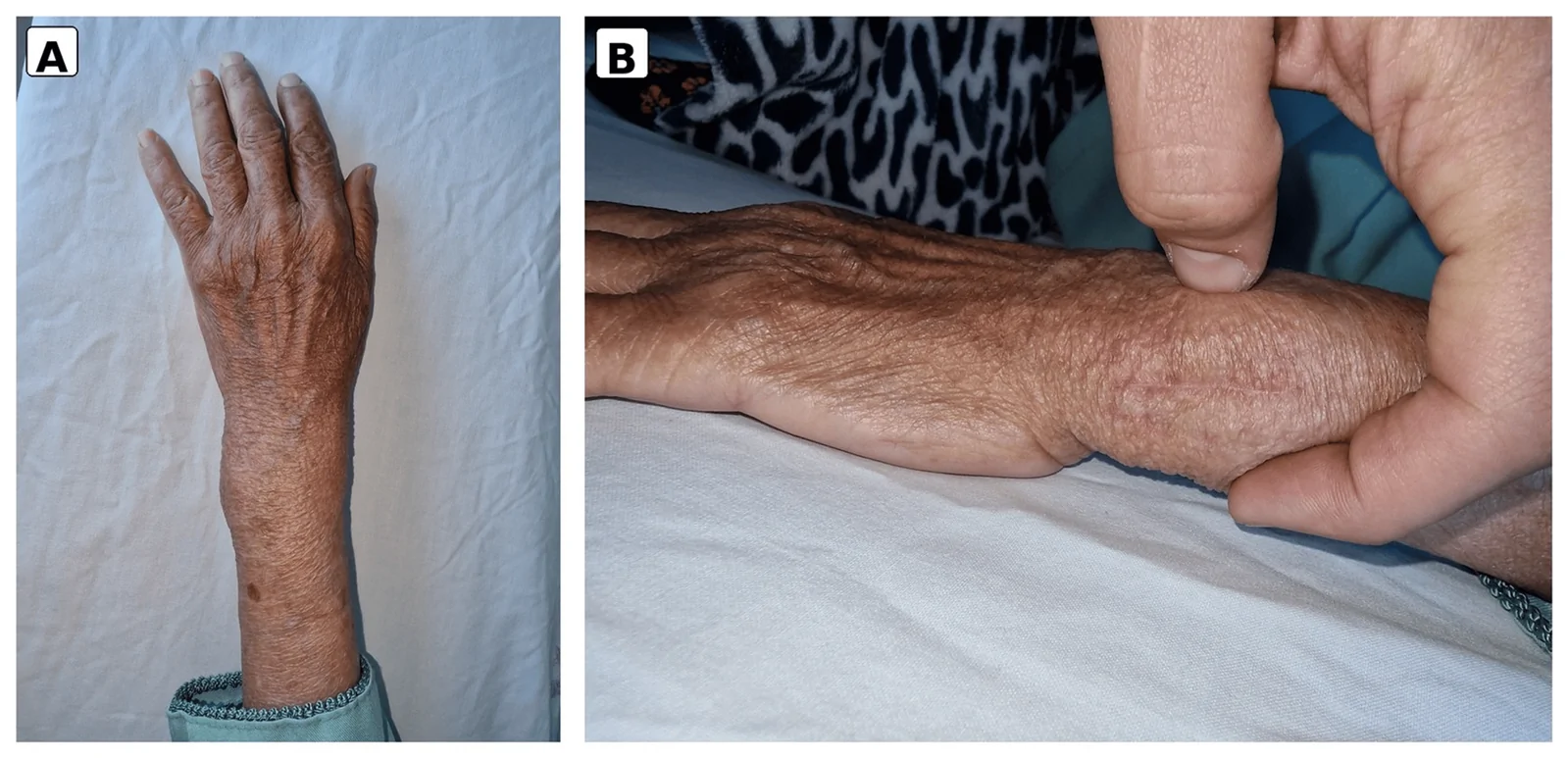
Preoperative clinical appearance of the left wrist (A) Dorsal view showing swelling of the ulnar border. (B) View during palpation showing a firm mass over the distal ulna.

Anteroposterior and lateral radiographs demonstrated a predominantly osteolytic, moderately expansile lesion involving the distal ulna from the epiphysis to the metaphysis, with cortical thinning and osseous expansion but no obvious periosteal reaction. The adjacent radius, carpal bones, and metacarpal bases were preserved, without frank cortical destruction or obvious joint-space involvement (Figure 2).

**Figure 2:**
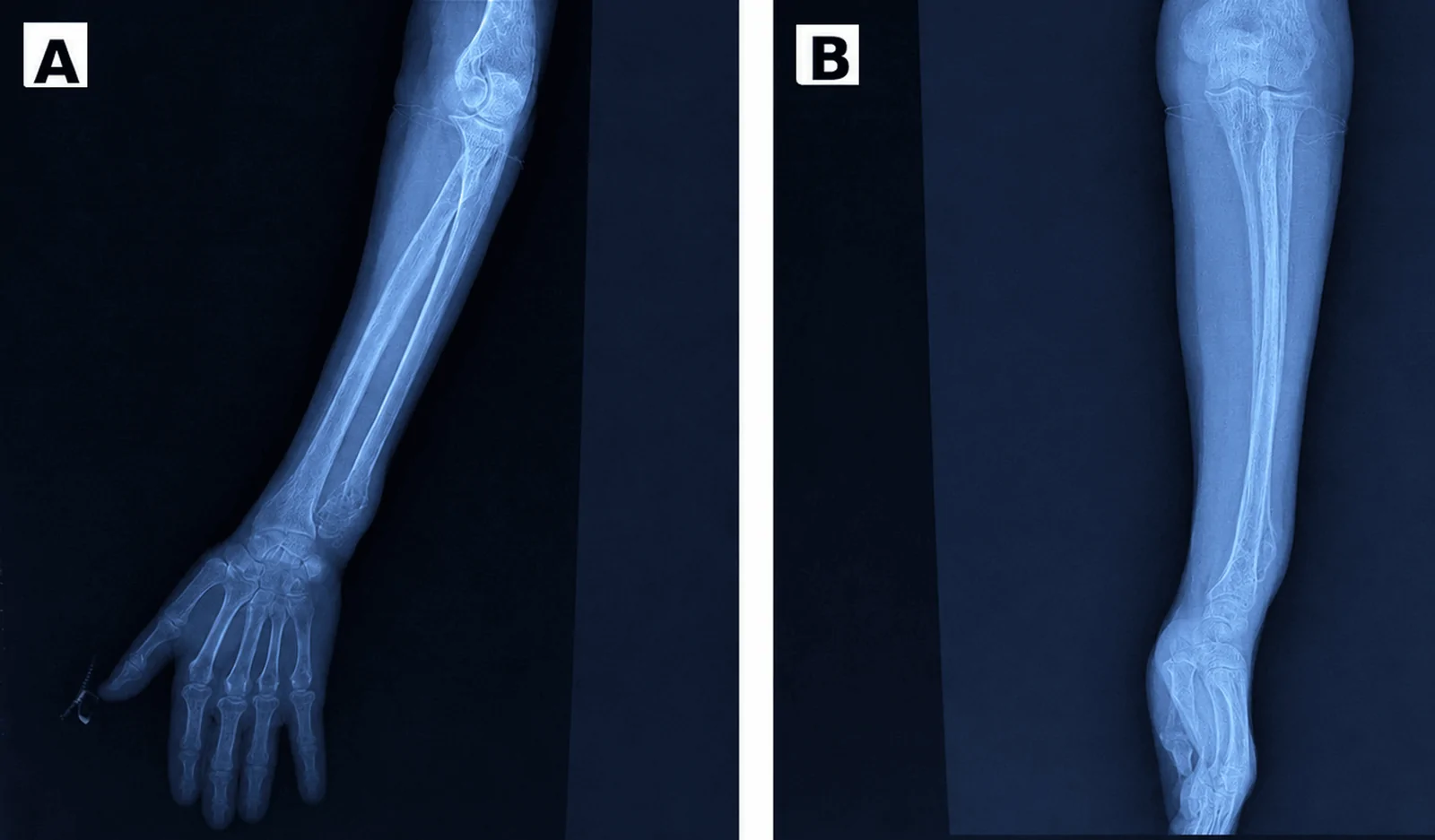
Preoperative radiographs of the left wrist (A) Anteroposterior view and (B) lateral view showing a predominantly osteolytic, moderately expansile lesion of the distal ulna, extending from the epiphyseal to the metaphyseal region, with cortical thinning and no obvious periosteal reaction.

Surgical biopsy showed a highly cellular lesion composed of mononuclear stromal cells and numerous relatively homogeneously distributed osteoclast-like multinucleated giant cells, without significant cytonuclear atypia or atypical mitoses, compatible with giant cell tumor (Figure 3).

**Figure 3:**
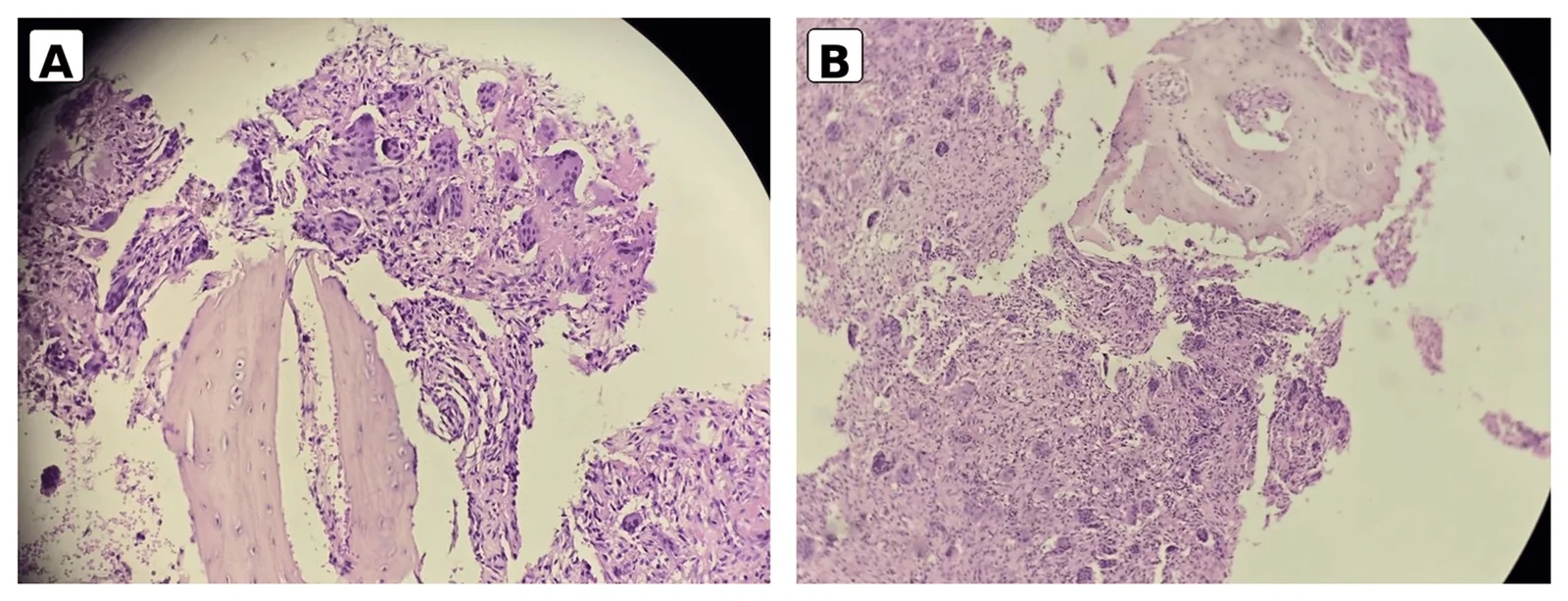
Histopathological examination of the surgical biopsy (A) Hematoxylin-eosin staining at ×100 magnification and (B) hematoxylin-eosin staining at ×40 magnification, showing a cellular lesion composed of mononuclear stromal cells and numerous osteoclast-like multinucleated giant cells, without significant atypia or atypical mitoses, compatible with a giant cell tumor of bone.

Given the background of chronic kidney disease on hemodialysis, the differential diagnosis of a brown tumor related to secondary hyperparathyroidism was considered. However, the biological workup, including serum calcium, phosphate, and parathyroid hormone levels, was within normal limits and did not support this diagnosis. In addition, the histological features were typical of a giant cell tumor. The extension workup, including chest imaging, did not reveal any secondary localization.

The patient underwent complete tumor excision according to a Darrach-type procedure. Under locoregional anesthesia and a pneumatic tourniquet, a longitudinal incision was made on the ulnar aspect of the wrist. Careful dissection allowed exposure of the distal ulna while preserving the surrounding neurovascular and tendinous structures. En bloc resection of the distal end of the ulna was performed, removing the tumor with macroscopically healthy margins. The macroscopic resection specimen is shown in Figure 4.

**Figure 4:**
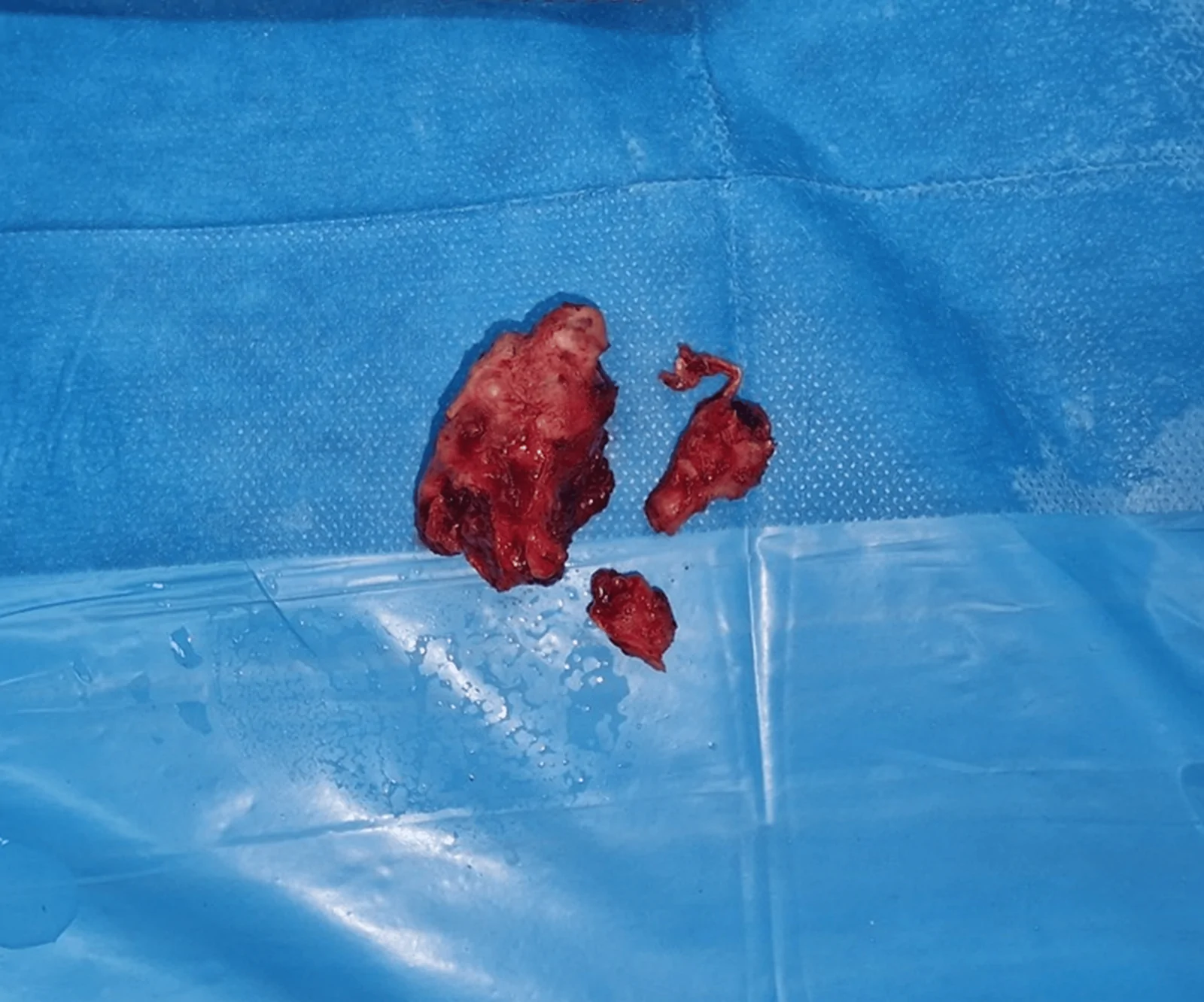
Macroscopic appearance of the distal ulna excision specimen showing the tumor segment after en bloc resection

To prevent postoperative instability of the ulnar stump, an additional soft-tissue stabilization procedure was performed. Tenodesis was performed using the flexor carpi ulnaris tendon to stabilize the ulnar stump and maintain wrist alignment. The ulnar stump was regularized to limit the risk of postoperative impingement. The flexor carpi ulnaris tenodesis technique used for stump stabilization is illustrated in Figure 5. After careful hemostasis, the wound was closed in layers over a drain (Figure 5).

**Figure 5:**
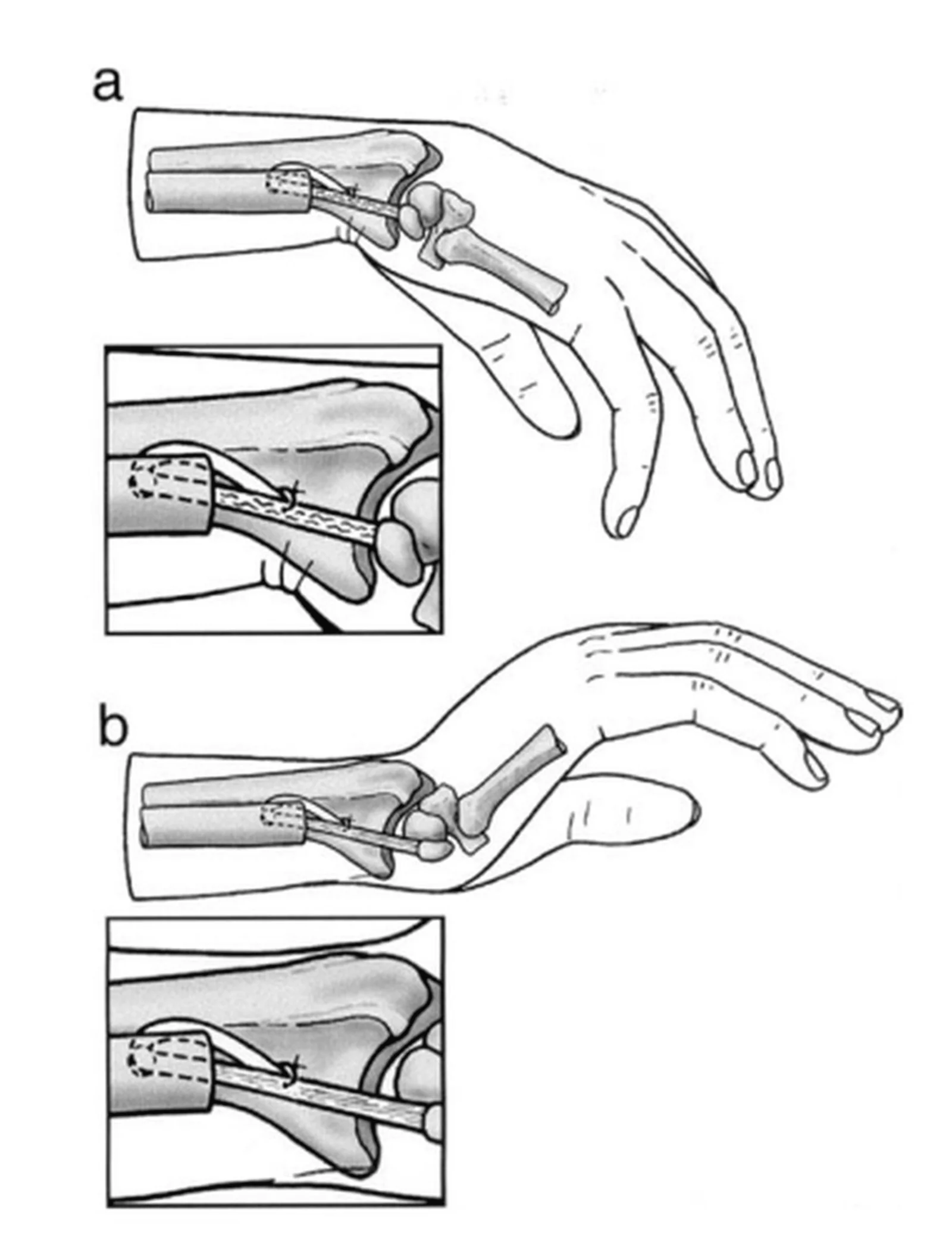
Schematic representation of flexor carpi ulnaris tenodesis for stabilization of the residual ulnar stump after distal ulna resection (a) The flexor carpi ulnaris tendon strip is routed through the residual ulna to stabilize the stump; the inset highlights the tendon fixation at the ulnar stump. (b) The reconstruction is shown from an alternate forearm position, with the inset demonstrating the tendon strip traversing the residual ulna. Reproduced with permission from Syed et al. [14]. © Springer-Verlag 2003. Permission was obtained through the Copyright Clearance Center/RightsLink.

Postoperative radiographs confirmed resection of the distal end of the ulna with satisfactory alignment and no immediate complication. The immediate postoperative radiographs are shown in Figure 6.

**Figure 6:**
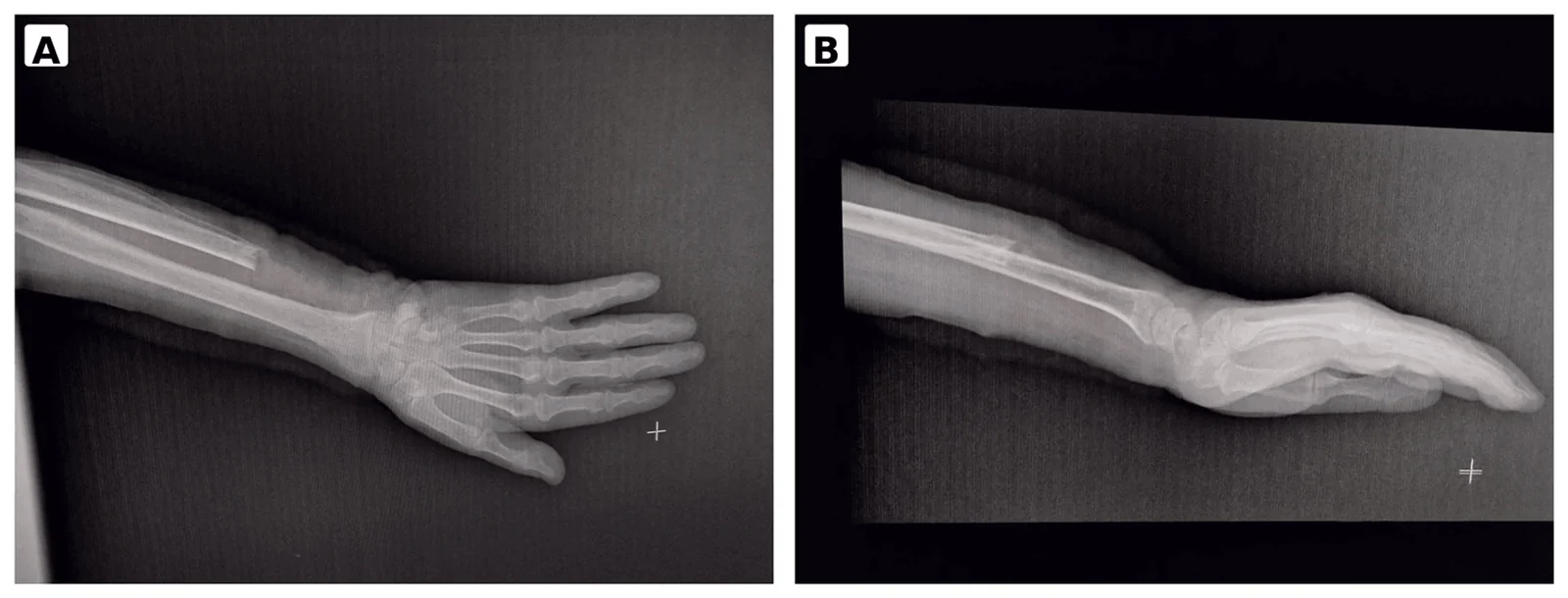
Immediate postoperative radiographs (A) Anteroposterior view and (B) lateral view after distal ulna resection, showing satisfactory wrist alignment without immediate complication.

Histopathological examination of the surgical specimen confirmed the diagnosis of giant cell tumor of bone. The lesion was characterized by a proliferation of mononuclear stromal cells associated with numerous osteoclast-like multinucleated giant cells, without significant cytonuclear atypia or atypical mitotic activity. These results were concordant with those of the preoperative biopsy and with the clinical and radiological data. Representative histopathological images of the excision specimen are shown in Figure 7.

**Figure 7:**
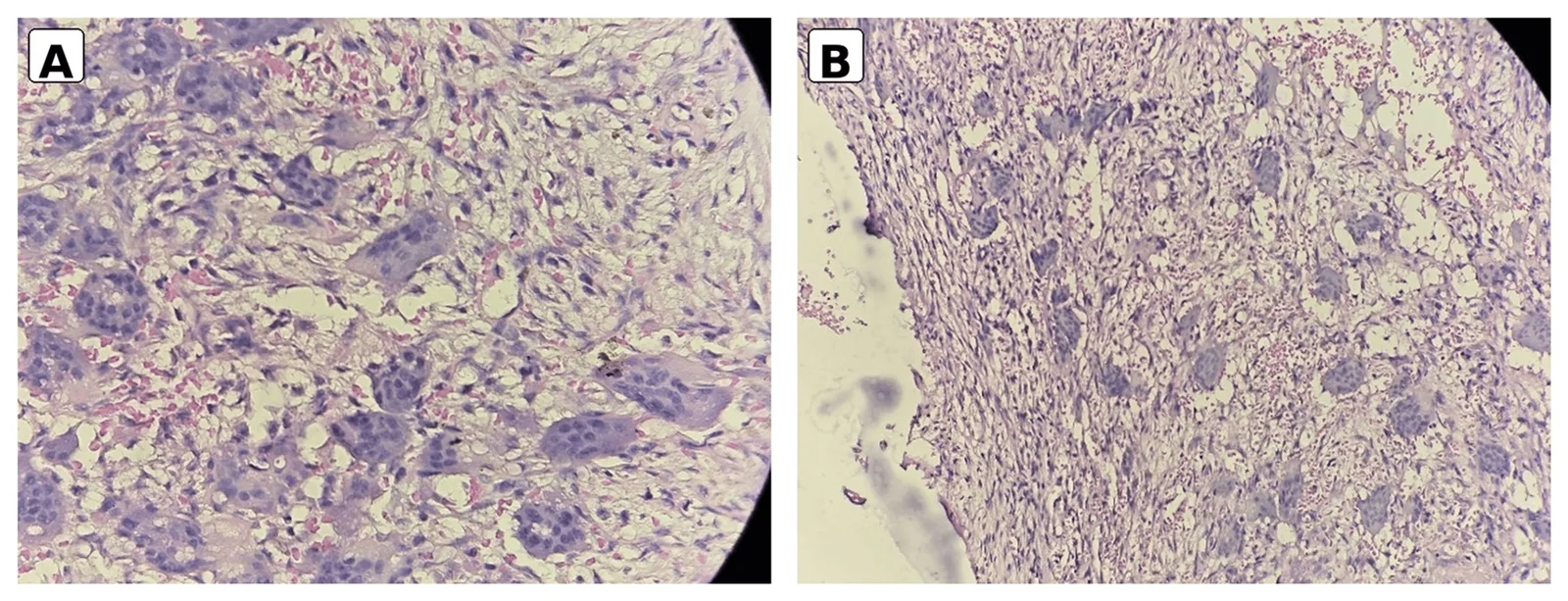
Histopathological examination of the excision specimen (A) Hematoxylin-eosin staining at ×200 magnification and (B) hematoxylin-eosin staining at ×100 magnification, confirming the giant cell tumor of bone, with mononuclear stromal cells and numerous osteoclast-like multinucleated giant cells.

Outcome and follow-up

The final available follow-up was eight months. Functional mobility was assessed clinically rather than using a validated patient-reported instrument. The patient demonstrated active functional wrist positions documented in Figure 8, indicating preserved clinical mobility compared with the painful and restricted motion documented preoperatively. The operative scar was quiet, and no early postoperative complications were reported. Quantitative goniometric range of motion, grip strength, pain scores, QuickDASH, and Mayo Wrist Score values were not prospectively recorded and therefore are not reported retrospectively.

**Figure 8:**
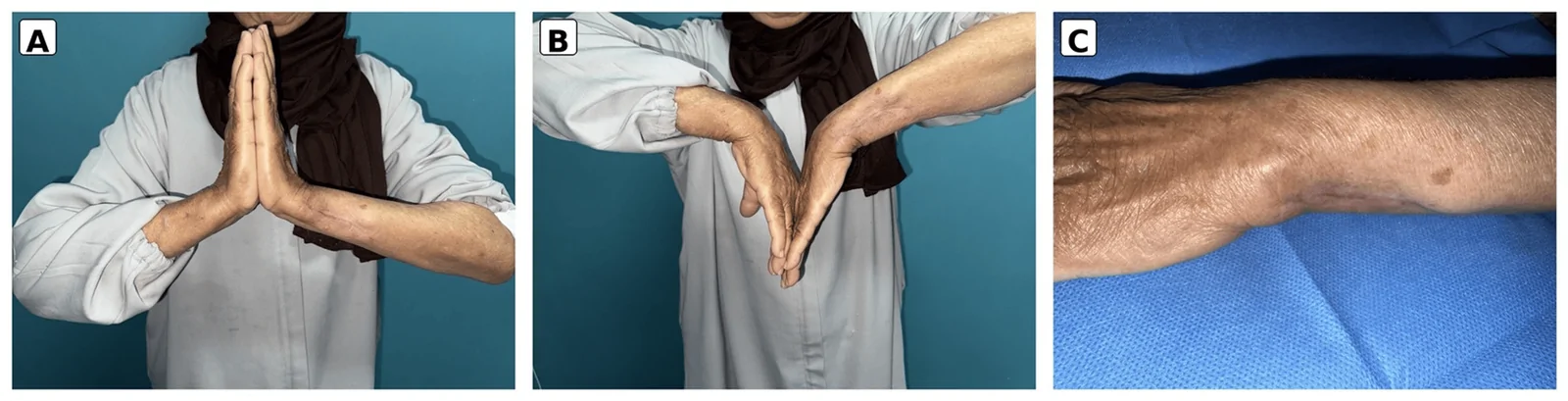
Clinical follow-up at eight months (A, B) Functional positions of the wrist showing preserved mobility. (C) Quiet operative scar, with no local inflammatory signs.

Eight-month radiographs showed a stable postoperative appearance without radiological evidence of recurrence or mechanical complication (Figure 9).

**Figure 9:**
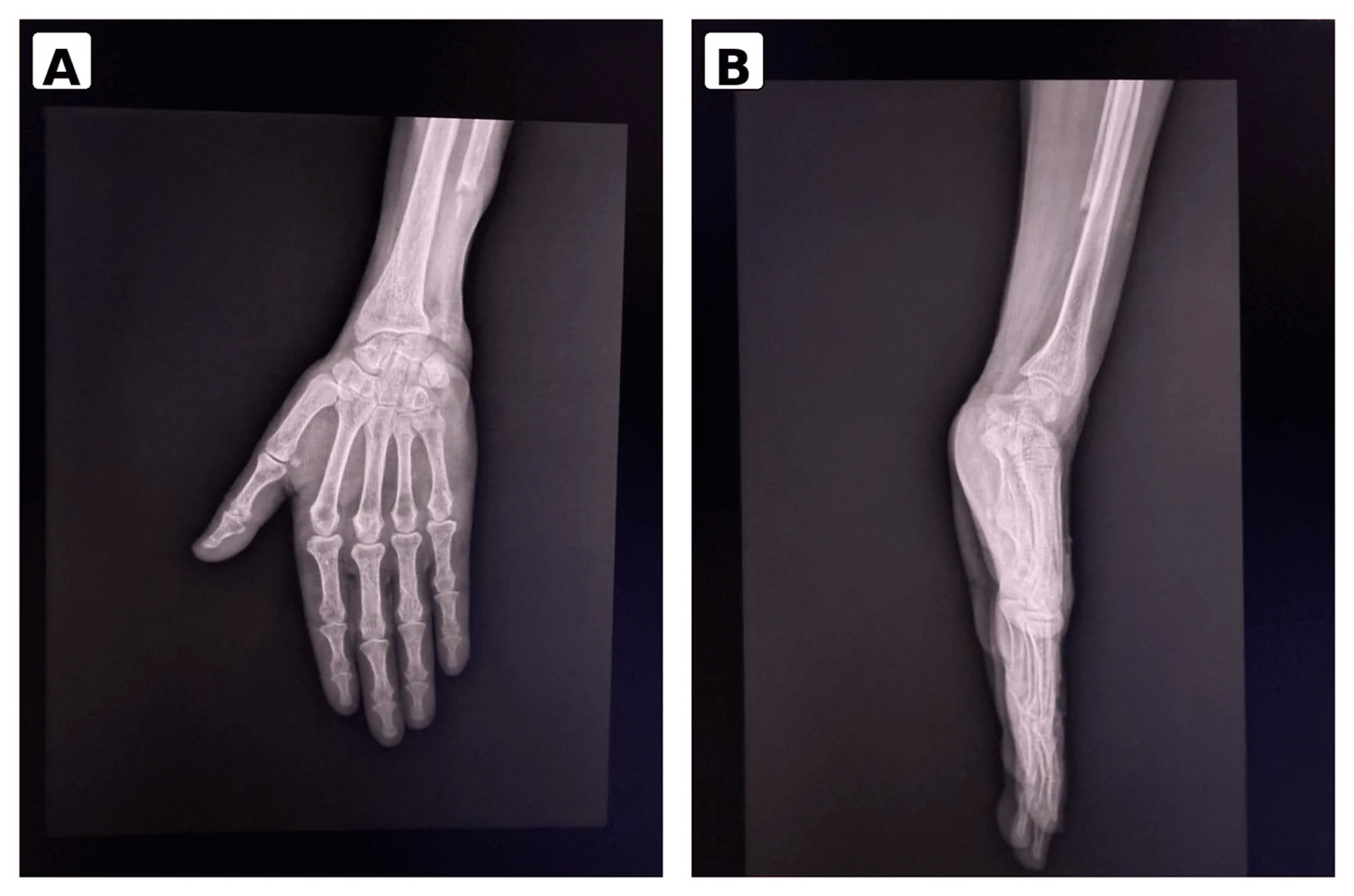
Follow-up radiographs at eight months (A) Anteroposterior view and (B) lateral view showing a stable postoperative appearance, with no radiological sign of recurrence or mechanical complication.

## Discussion

Giant cell tumor of bone classically affects young adults, with a predilection for long bones near joints [1-4]. Its occurrence in the distal ulna remains rare, which may delay diagnosis and broaden the differential diagnosis [5,6]. In our case, the advanced age of the patient and her hemodialysis background made the diagnosis particularly challenging.

In a hemodialysis patient, a giant-cell-rich expansile osteolytic lesion should raise the possibility of a brown tumor related to secondary hyperparathyroidism. These lesions may show radiological characteristics similar to those of a giant cell tumor, including expansile osteolysis, cortical thinning, and sometimes bone destruction. Histologically, the presence of multinucleated giant cells may also create diagnostic confusion [12,13]. The distinction therefore relies on rigorous correlation between clinical, biological, radiological, and histopathological data. In our case, the absence of abnormalities in serum calcium, phosphate, and parathyroid hormone levels, together with the typical histological features, supported the diagnosis of giant cell tumor.

The surgical management of giant cell tumors of the distal ulna remains debated because it must combine tumor control with preservation of wrist function. Intralesional curettage, possibly associated with local adjuvants, may preserve the distal radioulnar joint, but it exposes the patient to a higher risk of local recurrence, especially in aggressive lesions. In a multicenter study devoted to giant cell tumors of the distal ulna, Zhou et al. reported a significantly higher recurrence rate after curettage than after en bloc resection, with overall favorable functional outcomes after resection procedures [6]. Jamshidi et al. also suggested that curettage may be considered for limited grade 2 lesions, whereas distal ulna resection is preferable for more aggressive lesions with cortical involvement [9].

The distal ulna represents a particular anatomical location because its resection can often be performed with acceptable functional consequences compared with other periarticular locations. En bloc resection, including the Darrach procedure, has been described as an effective option for tumors of the distal ulna [7,8,10,11]. However, this technique may lead to painful instability of the ulnar stump, radioulnar convergence, decreased grip strength, and pain along the ulnar border of the wrist [11,15]. These complications explain the value of additional soft-tissue stabilization procedures.

In our case, flexor carpi ulnaris tenodesis was performed to improve the stability of the residual ulnar stump. Although many recent series mainly describe stabilization techniques using the extensor carpi ulnaris, flexor carpi ulnaris-based stabilization has also been described as a dynamic technique for distal radioulnar joint instability [16,17]. In an elderly, comorbid patient with limited functional demand, this strategy represents a pragmatic compromise between oncological safety and functional preservation while avoiding complex reconstructions that could increase operative time and morbidity.

The favorable early outcome observed in this case supports the value of distal ulna resection associated with soft-tissue stabilization in selected patients. Nevertheless, this observation has several limitations. The follow-up remains short; objective functional scores such as the QuickDASH [18] or Mayo Wrist Score [19] were not available, and no molecular confirmation, particularly testing for an H3F3A mutation, was performed. These limitations must be taken into account when interpreting the result. Prolonged clinical, radiographic, and thoracic follow-up remains essential because local recurrence may occur late and pulmonary metastases, although rare, have been reported in histologically benign giant cell tumors [3,4,6].

## Conclusions

In this 69-year-old patient on hemodialysis, clinicobiological and histopathological correlation supported the diagnosis of a distal-ulna giant cell tumor after a brown tumor secondary to hyperparathyroidism had been considered. En bloc distal-ulna resection with flexor carpi ulnaris tenodesis was followed, at eight months, by preserved clinical wrist mobility, stable radiographs, and no clinical or radiological recurrence. As this is a single case with short follow-up and no standardized functional score, these findings should be interpreted as an early patient-specific outcome rather than evidence of comparative efficacy. Continued surveillance and longer-term functional assessment remain necessary.
